# USP47 deficiency in mice modulates tumor infiltrating immune cells and enhances antitumor immune responses in prostate cancer

**DOI:** 10.1007/s00262-024-03730-5

**Published:** 2024-06-04

**Authors:** Qian-Lan Wang, Shun-Yuan Lu, Dan-Dan Xu, Jin-Xia Ma, Rui Guo, Lu Zhang, Ling-Yun Tang, Yan Shen, Chun-Ling Shen, Jin-Jin Wang, Ying-Li Wu, Li-Ming Lu, Zhu-Gang Wang, Hong-Xin Zhang

**Affiliations:** 1 Research Center for Experimental Medicine, State Key Laboratory of Medical Genomics, Shanghai Ruijin Hospital, Shanghai Jiao Tong University School of Medicine, Shanghai, 200025 China; 2 Shanghai Model Organisms Center, Shanghai, 201321 China; 3 Hongqiao International Institute of Medicine, Shanghai Tongren Hospital/Faculty of Basic Medicine, Department of Pathophysiology, Key Laboratory of Cell Differentiation and Apoptosis of the Chinese Ministry of Education, Shanghai Jiao Tong University School of Medicine, Shanghai, 200025 China; 4 Shanghai Institute of Immunology, Shanghai Jiao Tong University School of Medicine, Shanghai, 200025 China

**Keywords:** USP47, Tumor microenvironment, TIL, CTL

## Abstract

This study investigates the role of USP47, a deubiquitinating enzyme, in the tumor microenvironment and its impact on antitumor immune responses. Analysis of TCGA database revealed distinct expression patterns of USP47 in various tumor tissues and normal tissues. Prostate adenocarcinoma showed significant downregulation of USP47 compared to normal tissue. Correlation analysis demonstrated a positive association between USP47 expression levels and infiltrating CD8^+^ T cells, neutrophils, and macrophages, while showing a negative correlation with NKT cells. Furthermore, using *Usp47* knockout mice, we observed a slower tumor growth rate and reduced tumor burden. The absence of USP47 led to increased infiltration of immune cells, including neutrophils, macrophages, NK cells, NKT cells, and T cells. Additionally, USP47 deficiency resulted in enhanced activation of cytotoxic T lymphocytes (CTLs) and altered T cell subsets within the tumor microenvironment. These findings suggest that USP47 plays a critical role in modulating the tumor microenvironment and promoting antitumor immune responses, highlighting its potential as a therapeutic target in prostate cancer.

**Supplementary Information:**

The online version contains supplementary material available at 10.1007/s00262-024-03730-5.

## Introduction

Ubiquitin-specific protease 47 (USP47) is a member of the deubiquitinating enzyme (DUB) family, which consists of an N-terminal catalytic core domain and multiple ubiquitin-like domains (UBLs). The UBL domain facilitates protein-protein interactions, while the catalytic domain removes ubiquitin moieties from target proteins [1, 2]. USP47 is involved in various cellular processes, including DNA repair, cell cycle regulation, protein quality control, and signaling pathways. By deubiquitinating target proteins, USP47 modulates their stability, localization, and activity, including crucial proteins involved in DNA damage response and cell cycle progression like Polβ, SATB1, and RPS2 [2–5]. It also interacts with signaling molecules, such as those in the transforming growth factor-beta (TGF-β) and Wnt pathways, indicating its role in signal transduction [1, 6].

Accumulating evidence strongly suggests that dysregulation of USP47 contributes to the pathogenesis of several human diseases [7]. USP47 is expressed in various cancers and plays a significant role in tumor development and progression. Extensive research has shown its ability to promote cell proliferation, inhibit apoptosis, and enhance cancer cell invasiveness [8]. Additionally, USP47 impacts DNA repair mechanisms crucial for maintaining genomic stability and preventing cancer onset. Dysregulation of USP47 disrupts these repair pathways, leading to genomic instability and malignant transformation [9]. Notably, USP47 shows promise as a target for overcoming TKI resistance in chronic myelogenous leukemia (CML) and is implicated in protein aggregation and clearance pathways related to neurodevelopment and neurodegenerative diseases. Furthermore, studies have associated USP47 with diseases like myocardial infarction, intestinal inflammation and chronic kidney disease, highlighting its multifaceted functions [10–12].

Moreover, emerging evidence suggests a role for USP47 in immune regulation and inflammation. USP47 plays a crucial role in regulating NLRP3, a key member of the inflammasome complex involved in immune modulation and inflammatory responses [13]. It promotes NLRP3 assembly, inflammasome formation, and the release of pro-inflammatory cytokines like IL-1β and IL-18. These findings highlight the intricate interplay between USP47 and NLRP3, shedding light on their roles in inflammation regulation and immune responses. Recent studies have reported a positive correlation between USP47 expression and tumor-infiltrating Treg cells in colorectal and gastric cancer patients. USP47 is involved in maintaining Treg cell metabolic and functional homeostasis by suppressing YTHDF1-mediated translation of c-Myc. Targeted deletion of USP47 in Treg cells leads to inflammatory disorders and enhanced antitumor immune responses in mice [14]. USP47 has also been identified as a modulator of the antiviral innate immune response. It plays a crucial role in removing K63-linked polyubiquitin chains from TRAF3 and TRAF6, which ultimately leads to a reduction in type I IFN signaling [15].

To explore the physiological impact of USP47 on the antitumor immune response, we conducted a comprehensive analysis using a global *Usp47* knockout mouse model and a syngeneic RM-1 murine prostate tumor cell line on the C57BL/6 background. Assessing the effects of *Usp47* knockout on tumor growth and the tumor microenvironment, we made significant findings. The absence of host Usp47 resulted in notable growth retardation of the xenografts. Moreover, tumors grown in Usp47-deficient mice exhibited heightened levels of infiltrated immune cells, including granulocytes, NKT cells, and T cells. Notably, the lack of host USP47 led to an aberrant increase in CTL activation, suggesting a critical role for host USP47 in modulating antitumor immune cell homeostasis and participating in the anti-tumor process.

## Material and methods

### Cell culture

RM-1, a murine prostate cancer cell line, was cultured in Dulbecco’s modified Eagle’s medium (HyClone, USA) supplemented with 10% (v/v) fetal bovine serum (FBS, Gibco, USA) and antibiotics containing 10 U/mL of penicillin and 10 U/mL of streptomycin. All cells were routinely incubated at 37 ℃ in a humidified atmosphere of 5% CO2. *Usp47* knockout mice on C57BL/6 J background were obtained from MMRRC (Mutant Mouse Resource & Research Centers, USA). The *Usp47* genotype was determined using PCR analysis with the following primers: β-Geo (580 bp) 5′-CAAATGGCGATTACCGTTGA − 3′, 5′-TGCCCAGTCATAGCCGAATA-3′; Usp47 (907 bp) 5′-CTTCACCTGTTCAAATCC TCCG-3′, 5′-GTTCCTTTCTGTTCATA CCGATG-3′. The mice were housed in individual ventilated cages under specific pathogen-free conditions at the animal facility of the Research Center for Experimental Medicine, Ruijin Hospital, with adequate food. Male mice aged 6–8 weeks, including *Usp47* knockout homozygous (*Usp47*^*−/−*^) and wild-type (*Usp47*^+*/*+^) C57BL/6 J mice, were used for further analyses. All animal experiments were conducted in accordance with the approved protocols by the Animal Ethics Committee of Ruijin Hospital affiliated with Shanghai Jiao Tong University School of Medicine (Shanghai, China).

### Bioinformatic analysis

Prostate cancer sequencing data and related clinical data were collected from The Cancer Genome Atlas (TCGA) by the online official website (https://portal.gdc.cancer.gov/). Using the data downloaded above, we evaluated USP47 expression levels in 497 cancer samples. For the analysis results, a two-group t-test was performed. *p* < 0.05 indicated that USP47 was differentially expressed between tumor tissues and normal tissues. The correlation between USP47 expression and single infiltrating immune cells was imaged on the TIMER 2.0 online database (http://timer.cistrome.org/).

### Label of RM-1 cells with luciferase

Lentivirus loaded with firefly Luc gene was transfected into RM-1 cells in a 6 cm dish. After 18 h of transfection, the medium was replaced with fresh medium. Three days post-infection, the cells were cultured in complete medium with 4 μg/mL puromycin for 1 week. After characterizing the luciferase expression using the IVIS system (IVIS-Lumina series III, Norwalk, CT, USA), several single cell clones of RM-1-luc were identified for further study.

### In vivo tumor model and bioluminescence assay

Ten C57BL/6 wild-type and *Usp47*^*−/−*^ 6- to 8-week-old C57BL/6 mice were prepared. The RM-1 luc cells were subcutaneously injected into the outer area of the right forelimb of the mice at a concentration of 5 × 10^5^/100 μl. Mice were assessed for tumor growth every 2 days after injection. After the tumor diameter was approximately 3–4 mm around day 7, tumor growth was measured daily using a digital caliper. Tumor volume was calculated using the following formula: (length × width × width)/2. Simultaneously, live mouse imaging was performed periodically to measure fluorescence intensity. For imaging, mice were intraperitoneally injected with D-luciferin substrate (150 mg/kg), and after 7–10 min of injection, imaging was performed on the IVIS-Lumina series III imaging system. The fluorescence intensity values were subsequently analyzed using Living Image® software version 3.0.4 (Xenogen, Hopkinton, MA, USA). On day 16 of tumor growth, all mice were sacrificed for tumor stripping. All experiments were approved by the Animal Ethics Committee of Ruijin Hospital Affiliated to Shanghai Jiaotong University School of Medicine.

### Flow cytometry of tumor-infiltrating lymphocytes

After the RM-1 tumor was excised on ice, one part was separated for immunohistochemical staining, and the other part was physically ground with a 300-mesh filter, and the tumor cell suspension was passed through a 70 μm cell strainer. Red blood cell lysate (BD Bioscience, New York, NY, USA) was added. The mixtures were left at room temperature for 3 min and washed twice with PBS and resuspended in pre-cooled PBS. To analyze the proportions of immune cells, the isolated single cells were incubated with specific mouse antibodies in the dark at 4 °C for 30 min. The mouse-specific antibodies used included CD45, B220, CD3, CD8, CD69, CD44, CD62L, PD-1, CTLA4, CD11b, NK1.1, Gr-1, F4/80, and Zombie dye. The apoptosis of CD8 + tumor-infiltrating lymphocytes (TILs) was measured using the Annexin V Apoptosis Detection Kit (Thermo Fisher Scientific, Inc., Waltham, MA, USA). Flow cytometry analysis was performed using a FACSVerse flow cytometer (BD Bioscience, New York, USA), and the data were analyzed using FlowJo software. For a detailed list of all antibodies used, please refer to Table S1.

### Immunohistochemistry

Formalin (4%)-fixed, paraffin-embedded slides were prepared from the resected RM-1 luciferase tumor tissue above, with a thickness of approximately 5 μm. Slides were deparaffinized in xylene for 15 min and hydrated through a concentration gradient of ethanol to water. The endogenous peroxidase activity was blocked by placing slides in 3% H_2_O_2_ methanol for 10 min at room temperature. Subsequently, sections were incubated with blocking buffer (10% goat serum, 0.5% Triton X-100 in 1 × PBS) for 30 min. And then the dilution of Ki67 (1:200) and Caspase-3 (1:200) were applied to the slides and incubated overnight at room temperature. Next day, slides were incubated with HRP-conjugated secondary antibody for 1 h at room temperature. Subsequent development for 10 min in 3,3-diaminobenzidine tetrahydrochloride (DAB) containing 0.002% hydrogen peroxide produces a brown stain. Finally, counterstaining was performed with hematoxylin. Images were captured using a Nikon microscope (Tokyo, Japan), and the staining intensity was quantified using Image Pro Plus 6.0 software (Media Cybernetics, Inc., Maryland, United States).

### Statistical analysis

One-way or two-way analysis of variance (ANOVA) was used to analyze the significant differences among multiple groups. The data are expressed as the mean ± Standard Error of the Mean (SEM) for continuous variables and frequency (percentage) for categorical variables, unless otherwise described in the figure legends. For all statistical tests, *p*-values < 0.05 were considered to be statistically significant. All statistical tests were performed using GraphPad Prism 9.0 (GraphPad Software Inc.).

## Results

### USP47 was associated with the infiltrated immune cells in prostate cancer

**Fig. 1:**
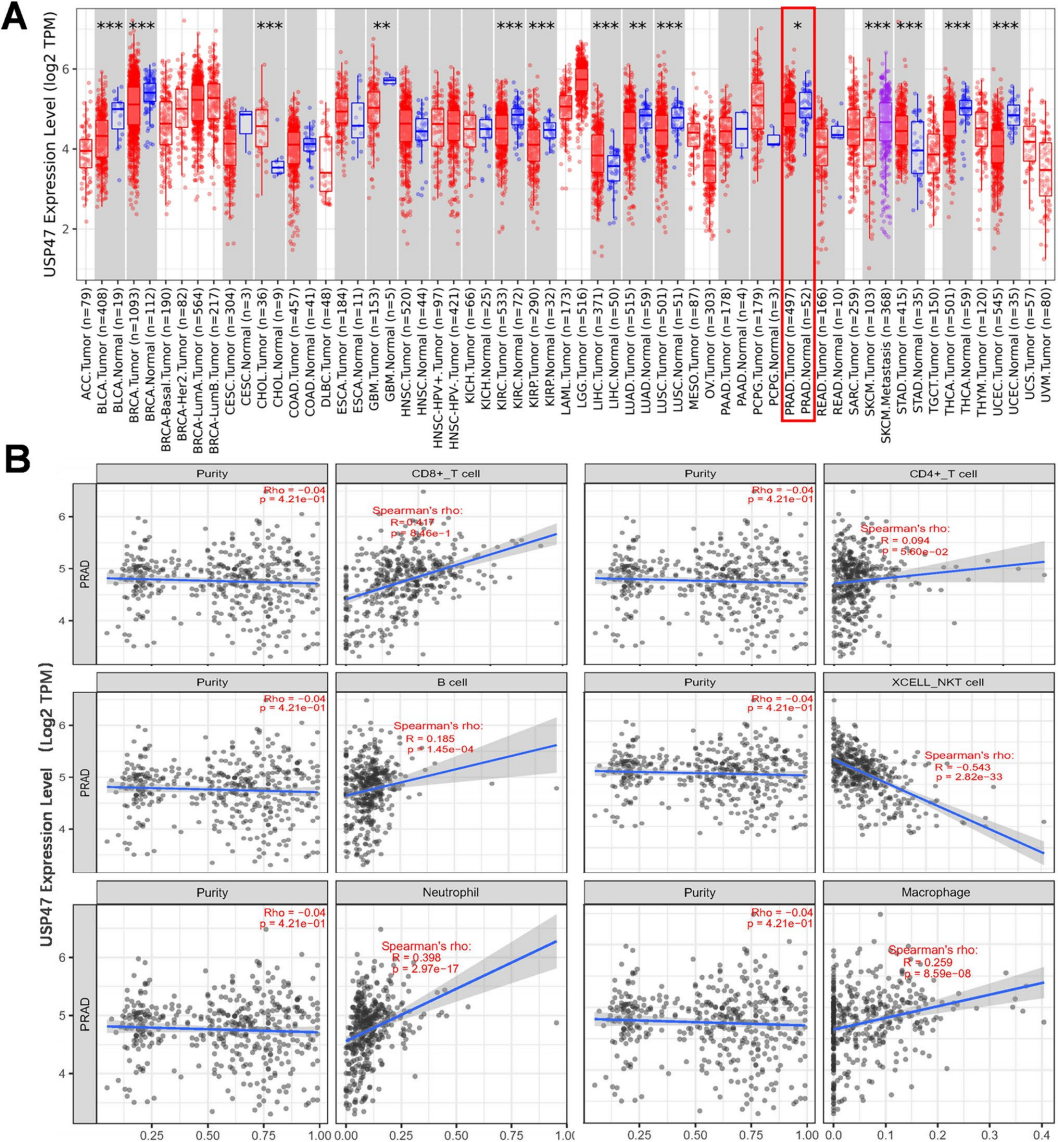
USP47 expression levels in human cancers and its correlation with immune infiltration in prostate cancer. **A** The expression levels of USP47 across various tumor types were determined using the TIMER web server based on TCGA data (*p < 0.05, **p < 0.01, ***p < 0.001).** B** Positive correlations of USP47 expression with immune cell subpopulations in the tumor microenvironment of prostate cancer (PRAD)

We conducted an analysis of USP47 expression in various tumors and normal tissues using data from the TCGA database, as determined by TIMER. The results unveiled distinct expression patterns of USP47 across different tumor tissues and normal tissues. Some tumor tissues displayed higher expression levels of USP47 compared to normal tissues, while others exhibited lower expression levels (Fig. 1A). These findings suggest that USP47 may play diverse roles in the development, progression, and anti-tumor responses of different tumors. To further explore the potential role of USP47 in the anti-tumor response, we focused on prostate adenocarcinoma (PRAD) for additional analysis. Our findings indicate a significant downregulation of USP47 expression in prostate cancer when compared to normal tissue. To evaluate the association between USP47 and infiltrating immune cells in PRAD, we analyzed the correlation between USP47 expression and six types of infiltrating immune cells: CD8^+^ T cells, CD4^+^ T cells, B cells, NKT cells, neutrophils, and macrophages (Fig. 1B). The results revealed a significantly positive correlation between USP47 expression levels and the levels of infiltrating CD8^+^ T cells, neutrophils, and macrophages, while indicating a negative correlation with NKT cells. These data accentuate the differential expression patterns of USP47 in various tumors and normal tissues, suggesting its potential involvement in tumor development, progression, and anti-tumor responses. Specifically, in prostate cancer, USP47 expression is significantly downregulated compared to normal tissue, and its expression levels correlate positively with infiltrating levels of certain immune cells. The interplay between tumor cells and infiltrating immune cells is a complex dynamic that can impact the outcome of cancer progression. The functionality of a gene frequently hinges on its cellular context and the specific biological milieu it inhabits. Our unpublished data indicate that *Usp47* gene knockout in mouse RM-1 prostate cancer cells can lead to accelerated tumor growth. In this current study, we focused on the role of USP47 in the anti-tumor immune response of prostate cancer.

### RM-1 tumor growth was inhibited in Usp47 knockout mice

**Fig. 2:**
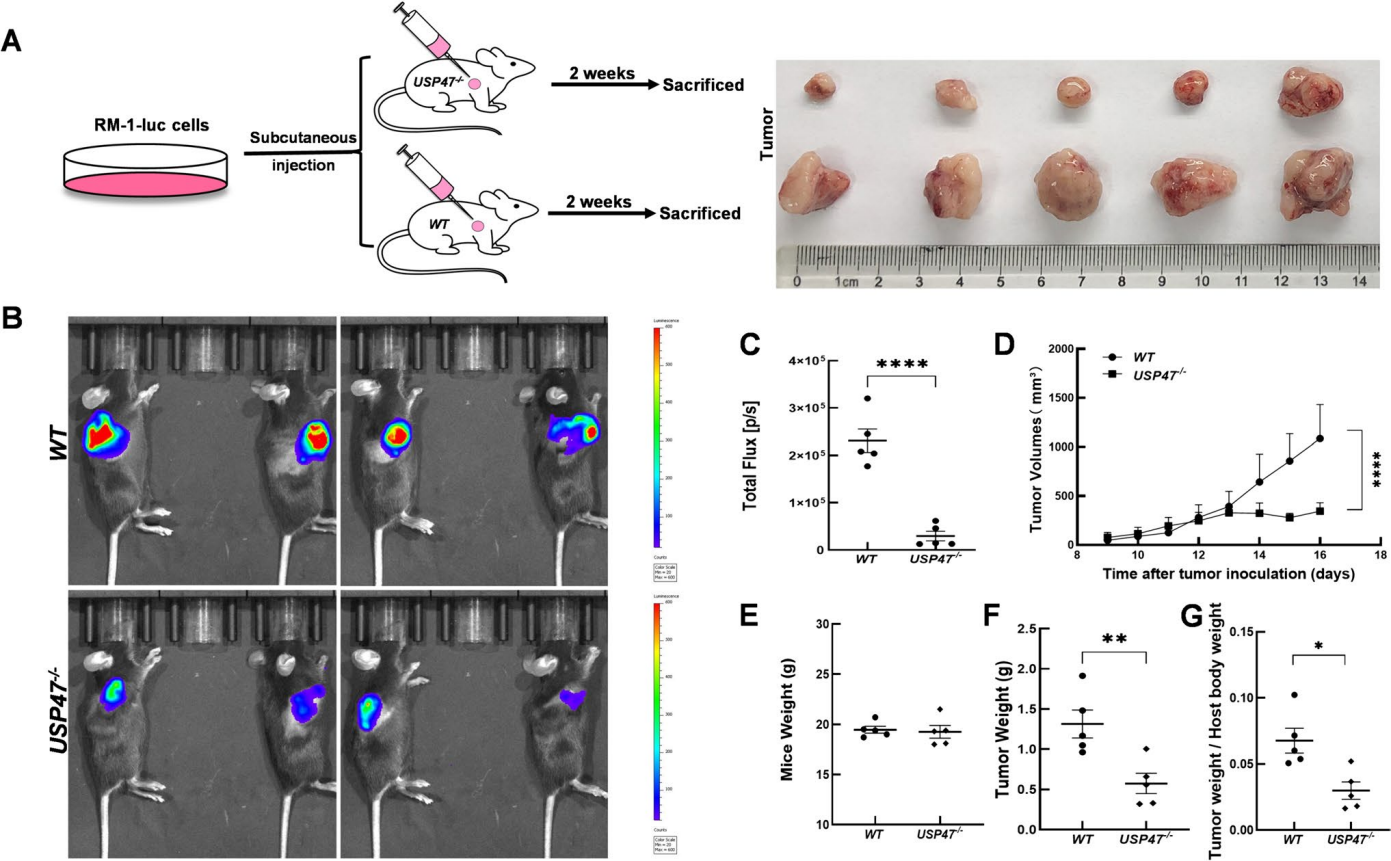
Target deletion of host *Usp47* inhibits RM-1-luc tumor growth in C57BL/6 J mice. RM-1-luc murine prostate cancer cells were directly injected into WT and *Usp47* knockout mice (n = 5/group). **A** Schematic diagram of the tumor-bearing mouse model and representative tumor photograph. **B** In vivo fluorescence imaging for detection of RM-1-luc tumors. **C** Quantification of tumor luminescence intensity. **D** Measurement of tumor volume. **E–F** Analysis of mice weight, tumor weight, and ratio of tumor weight to host body weight. The results are presented as mean ± SEM from three independent experiments. Statistical comparisons between groups were performed using Student’s t-test. *p < 0.05; **p < 0.01, ****p < 0.0001

**Fig. 3:**
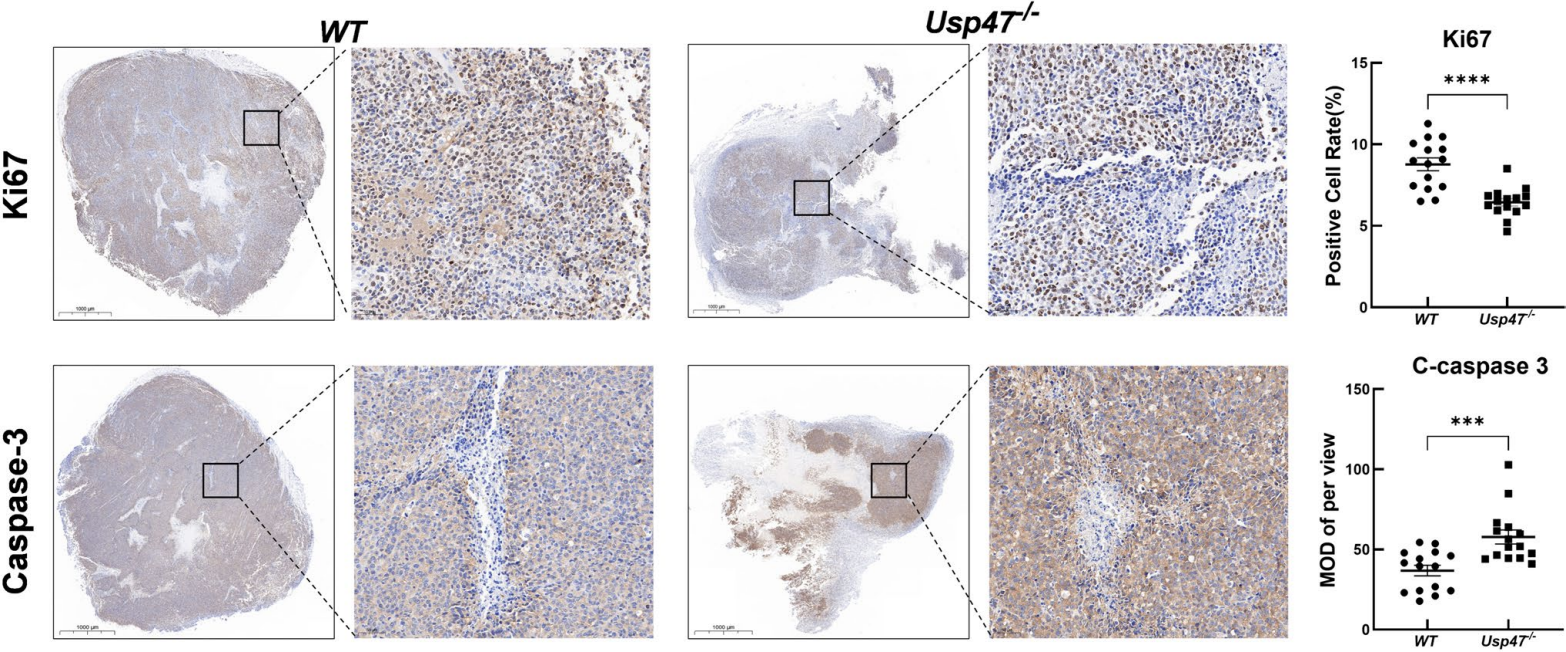
Impact of host *USP47* deletion on RM-1 tumor cell proliferation and apoptosis. Cell proliferation and apoptosis in the tumor tissue were assessed using immunohistochemistry for Ki67 and cleaved caspase-3, respectively (n = 3/group). The scale bars represent 2000 μm (left) and 50 μm (right). The average optical density of Ki67 and cleaved caspase-3 staining was quantified using Image Pro Plus 6.0 software (Media Cybernetics, Inc.). The data are presented as mean ± SEM. MOD: mean optical density. Statistical significance is indicated as ***p < 0.001, ****p < 0.0001

To investigate the impact of host USP47 deficiency on tumor growth, we conducted a comprehensive study using both wild-type and *Usp47* knockout mice. The mice were injected with RM-1-luciferase murine prostate cancer cells, and the subsequent tumor growth was closely monitored over time. The results provided compelling evidence that the absence of Usp47 in mice led to a significantly slower growth rate of the tumors compared to the WT mice. In addition to observing the differences in tumor growth rate, we also assessed the tumor weight and the tumor/body weight ratio in both groups of mice. Remarkably, we found a notable decrease in both parameters in the *Usp47* knockout mice (*Usp47*^*−/−*^) (Fig. 2). This finding strongly suggested that the absence of host USP47 had a profound impact on the overall tumor burden. To further investigate the underlying mechanisms responsible for the reduced tumor burden in *Usp47* knockout mice, we turned our attention to examining the levels of cell proliferation and apoptosis in the tumor tissues. To assess cell proliferation, we employed immunohistochemical staining for Ki-67, a well-established marker for proliferating cells. The results, as depicted in Fig. 3, clearly indicated a significant decrease in the proliferation of tumor xenografts lacking host USP47. Conversely, when we evaluated the levels of apoptosis in the tumor tissues, we made an intriguing observation. The expression of cleaved caspase 3, a key marker of apoptosis, was notably increased in the tumor xenografts of *Usp47*^*−/−*^ mice. This finding strongly suggested that the absence of USP47 facilitated a relatively higher level of apoptosis in the tumor cells. Taken together, our findings provide compelling evidence that host USP47 deficiency significantly alters tumor growth dynamics in mice Fig. 2.

### Usp47^−/−^ mice show tumors with increased tumor-infiltrated immune cells

**Fig. 4:**
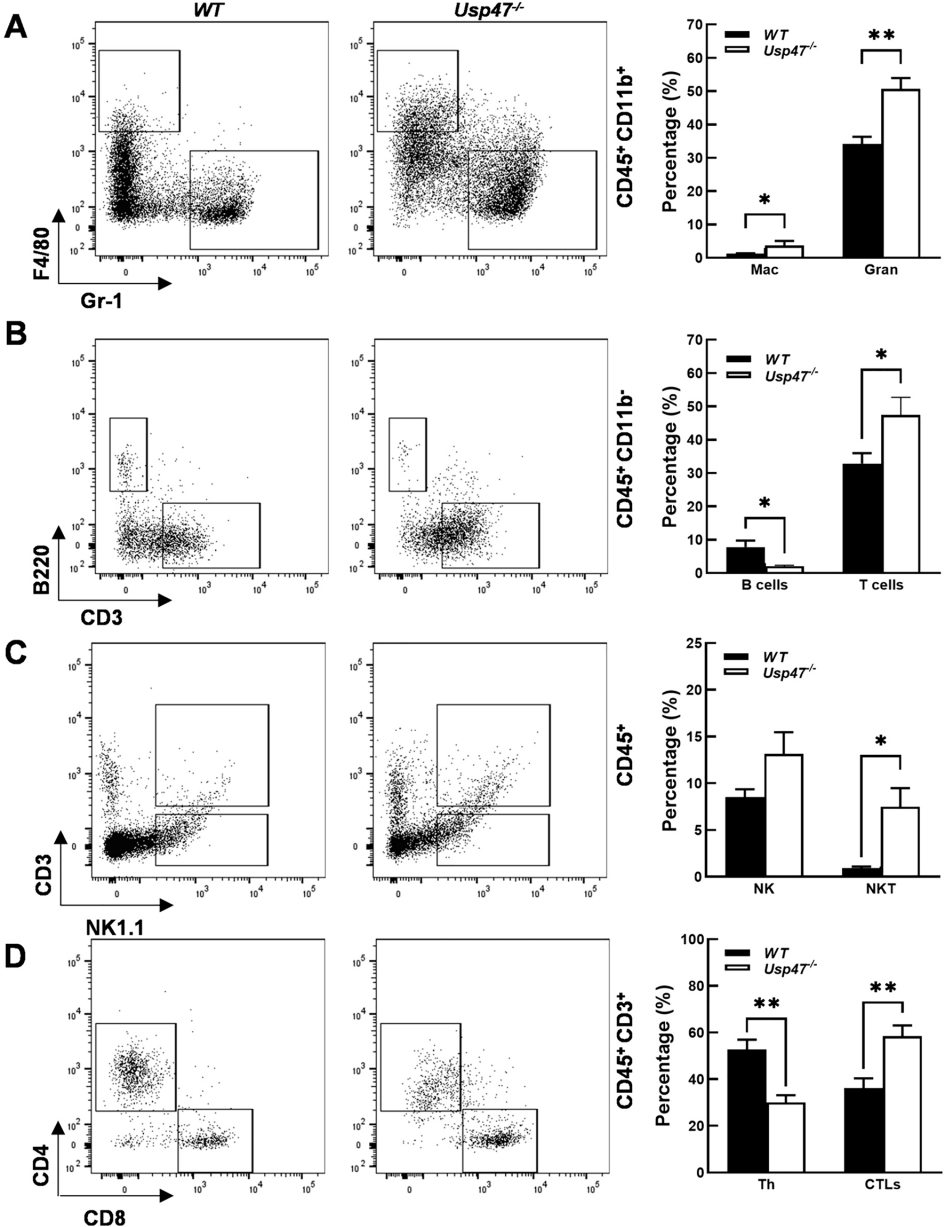
Analysis of tumor-infiltrating immune cells in tumors of WT and *Usp47* knockout mice using flow cytometry. **A** Macrophages (CD45^+^CD11b^+^F4/80^+^) and neutrophile granulocytes (CD45^+^CD11b^+^Gr-1^+^); **B** B cells (CD45^+^B220^+^) and T lymphocytes (CD45^+^CD3^+^); **C** NK cells (CD45^+^NK1.1^+^) and NKT cells (CD45^+^NK1.1^+^CD3^+^); **D** Th cells (CD45^+^CD3^+^CD4^+^) and CTLs (CD45^+^CD3^+^CD8^+^). Representative results from three independent experiments are shown. The data are presented as mean ± SEM. Statistical significance was determined using a two-sided unpaired Student's t-test. *p < 0.05; **p < 0.01. n = 5/group

To investigate whether the accelerated tumor growth observed in *Usp47*^*−/−*^ mice is attributed to changes in the tumor microenvironment (TME), we conducted an analysis of the type and quantity of infiltrating immune cells in tumors derived from *Usp47*^*−/−*^ and wild-type mice. Seventeen days after the injection of RM-1 cells, we performed flow cytometry analysis to assess the percentage of viable CD45^+^ immune cells infiltrating the tumors. The results revealed that the overall percentage of infiltrated immune cells was comparable between tumors from *Usp47*^*−/−*^ and WT mice. This suggests that the absence of Usp47 in the host does not significantly affect the overall recruitment of immune cells into the tumor. However, our investigation did uncover intriguing alterations in the composition of specific leukocyte populations within the TME upon *Usp47* knockout. Notably, the targeted deletion of host *Usp47* resulted in an increased percentage of tumor-infiltrating neutrophil granulocytes (CD45^+^CD11b^+^Gr-1^+^), macrophages (CD45^+^CD11b^+^F4/80^+^), natural killer (NK) cells (CD45^+^NK1.1^+^), natural killer T (NKT) cells (CD45^+^NK1.1^+^CD3^+^), and T cells (CD45^+^CD3^+^) (Fig. 4A-C). These findings suggest that USP47 may play a role in modulating the recruitment or function of specific immune cell populations within the TME. The increased presence of neutrophil granulocytes, macrophages, NK cells, NKT cells, and T cells in *Usp47*^*−/−*^ tumors could potentially contribute to the observed reduction in tumor growth and enhanced apoptosis.

### Usp47^−/−^ mice show tumors with increased activation of tumor-infiltrated CTLs

**Fig. 5:**
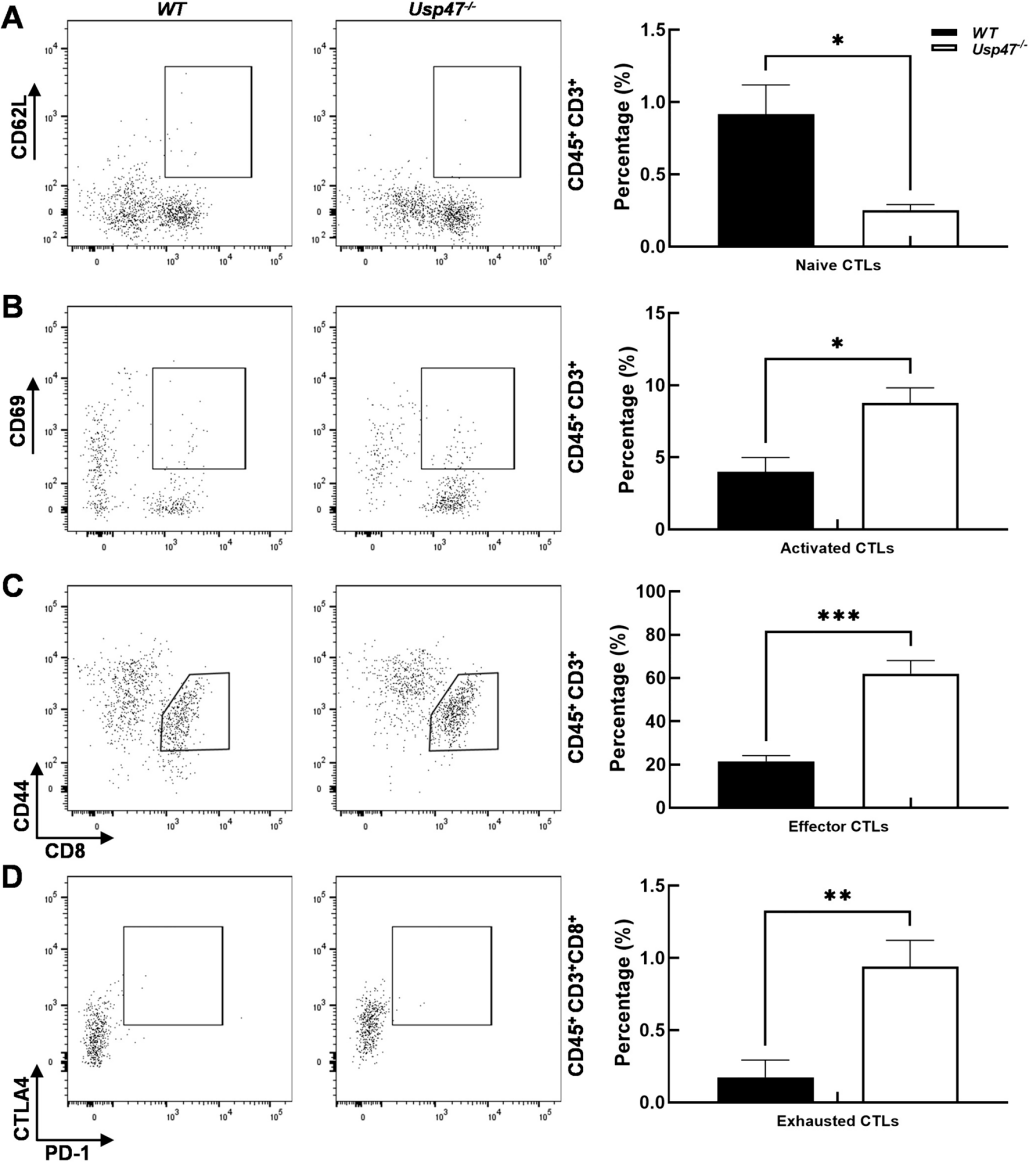
Enhanced activation of CD8 + T cells upon host *Usp47* deletion. **A** Naïve CTLs (CD45^+^CD3^+^CD8^+^CD44^–^CD62L^+^), **B** activate CTLs (CD45^+^CD3^+^CD8^+^CD69^+^), **C** effector CTLs (CD45^+^CD3^+^CD8^+^CD44^+^CD62L^−^), **D** exhausted CTLs (CD45^+^CD3^+^CD8^+^PD-1^+^CTLA4^+^). The frequencies (%) of CTLs at different stages in tumor-infiltrating lymphocytes (TILs) are shown in the bar graphs as mean ± SEM. Statistical significance was determined using unpaired two-tailed t-tests. *p < 0.05; **p < 0.01. n = 5/group

T cells play crucial roles in immune surveillance, tumor clearance, and immunotherapy against malignant tumors. In our study, we delved deeper into the impact of Usp47 deficiency on T cell populations within the tumor microenvironment. Specifically, we examined the infiltration of CD8^+^ cytotoxic T lymphocytes (CTLs) and CD4^+^ helper T cells (Th cells) in *Usp47*^*−/−*^ mice compared to wild-type (WT) mice. Our analysis revealed a significant increase in the percentage of tumor-infiltrating CD8^+^ CTLs (CD45^+^CD3^+^CD8^+^) in *Usp47*^*−/−*^ mice, indicating a potential enhancement in the antitumor immune response mediated by CTLs. Conversely, Usp47 deficiency led to a decrease in CD4^+^ Th cells (CD45^+^CD3^+^CD4^+^), suggesting a potential impairment in the helper T cell-mediated immune response (Fig. 4D). Furthermore, we observed significantly lower apoptosis levels of tumor-infiltrating CTLs in *Usp47*^*−/−*^ mice compared to wild-type mice (Fig. S1). These findings highlight the critical role of Usp47 in regulating the survival and apoptosis of tumor-infiltrating CTLs, underscoring its biological significance in modulating the antitumor immune response within the tumor microenvironment. Additionally, our investigation into CTL subsets revealed significant increases in activated CTLs (CD45^+^CD3^+^CD8^+^CD69^+^), effector CTLs (CD45^+^CD3^+^CD8^+^CD44^+^CD62L^−^), and exhausted CTLs (CD45^+^CD3^+^CD8^+^PD-1^+^CTLA4^+^), along with a decrease in naïve CTLs (CD45^+^CD3^+^CD8^+^CD44^−^CD62L^+^) (Fig. 5) in *Usp47*^*−/−*^ mice. These findings provide compelling evidence that Usp47 may play a critical role in the activation process of CTLs. The dysregulation of CTL responses within the TME due to Usp47 deficiency could impact the efficacy of antitumor immune responses and contribute to the observed changes in tumor growth dynamics in *Usp47*^*−/−*^ mice. Moreover, we assessed the impact of *Usp47* knockout on myeloid-derived suppressor cells (MDSCs), a cell population with significant immunosuppressive function (Fig. S2). We observed a marked reduction in the proportion of polymorphonuclear MDSCs (PMN-MDSCs) that play a critical role in mediating the immunosuppression of antigen-specific T cell responses, in the context of Usp47 deficiency. This finding provides a plausible, though partial, explanation for the enhancement of CTL activation caused by target deletion of host *Usp47* gene.

## Discussion

The tumor microenvironment is a complex ecosystem comprising diverse cell populations, extracellular components, and a vascular network [16, 17]. Within this intricate network, constant interactions and influences occur, providing support to tumor cells and facilitating their migration. Extensive research has demonstrated that the TME not only plays a crucial role in cancer progression and evolution but also significantly impacts the efficacy of various therapeutic approaches. Consequently, researchers have shown a growing interest in targeting the tumor microenvironment as a promising avenue for developing novel therapeutic strategies against malignant cancers [18, 19]. Therefore, the identification of novel modulators that can influence the TME is of utmost importance. In this study, we aimed to investigate the potential role of USP47, a deubiquitinating enzyme, in modulating the tumor microenvironment. Through bioinformatic analysis, we observed distinct expression patterns of USP47 across various tumors and normal tissues, indicating its involvement in different tumor types. Importantly, our findings demonstrated a significant downregulation of USP47 expression in prostate cancer compared to normal tissue. Moreover, we discovered a positive correlation between USP47 expression levels and the infiltration of CD8^+^ T cells, neutrophils, and macrophages in PRAD, suggesting its potential in regulating the dynamics of tumor cells within the TME. These results shed light on the possible role of USP47 in influencing the TME and provide a foundation for further exploration of its functional significance in cancer. However, we cannot solely rely on this correlation to determine whether the level of gene expression affects the intensity of the anti-tumor immune response. This prompts us to attempt to comprehensively understand the role of USP47 in the host's anti-tumor immune response.

To further explore the functional significance of USP47 in the TME, we employed a global *Usp47* knockout mouse model. Using a syngeneic RM-1 murine prostate cancer cell line, we examined the impact of host USP47 on tumorigenesis. The results demonstrated a significant inhibition of RM-1 tumor growth in vivo upon the absence of host Usp47. This observed phenotype can be attributed to two key factors: a decrease in tumor cell proliferation and an increase in apoptosis, both of which are consequences of host *Usp47* deletion. The tumor microenvironment plays a crucial role in regulating tumor cell proliferation and apoptosis, with a particular emphasis on the impact of immune cells in antitumor responses. Within the TME, immune cells exert a dual effect on tumor cell fate [20]. On one hand, cytotoxic immune cells, such as cytotoxic T lymphocytes and natural killer cells, directly target and eliminate tumor cells through recognition of tumor-specific antigens and release of cytotoxic molecules. These immune cells induce apoptosis in tumor cells, thereby inhibiting their proliferation and growth. On the other hand, certain immune cells, such as regulatory T cells and tumor-associated macrophages (TAMs), can promote tumor cell survival and proliferation by creating an immunosuppressive and pro-tumorigenic microenvironment. Tregs suppress the activity of effector immune cells, including CTLs, while TAMs secrete growth factors and cytokines that support tumor cell growth and angiogenesis. Therefore, the balance between effector immune cells and immunosuppressive cells within the tumor microenvironment determines the outcome of antitumor immune responses [21]. The analysis of infiltrating immune cells in the tumor xenografts of *Usp47*^*−/−*^ mice revealed an increased presence of specific immune cell populations, including neutrophil granulocytes, macrophages, NK cells, NKT cells, and T cells. This suggests that USP47 may play a crucial role in modulating the recruitment or function of these immune cells within the tumor microenvironment. The altered immune cell composition observed in *Usp47* knockout tumors may contribute to the observed changes in tumor growth dynamics and the immune response.

Cytotoxic T lymphocytes play a pivotal role in the immune response against tumors, exerting their cytotoxic function to eliminate cancer cells [22, 23]. As effector cells of the adaptive immune system, CTLs are capable of recognizing and destroying tumor cells through the recognition of tumor-specific antigens presented on major histocompatibility complex class I molecules. CTLs are generated through a complex process of T cell development and activation. Antigen-presenting cells, such as dendritic cells, capture tumor antigens and present them to naïve T cells in lymphoid organs. This interaction, coupled with co-stimulatory signals, leads to the activation and differentiation of T cells into CTLs. Once activated, CTLs undergo clonal expansion to generate a large population of effector cells that can infiltrate the tumor site. Activated CTLs possess several mechanisms to eliminate tumor cells. They release cytotoxic molecules, such as perforin and granzymes, which induce apoptosis in target cells. Additionally, CTLs can express cell surface molecules and trigger apoptosis in tumor cells through receptor-mediated pathways. Moreover, CTLs produce cytokines, such as interferon-gamma (IFN-γ), which can further enhance the antitumor immune response by promoting inflammation and activating other immune cells [24, 25]. In our study, we found that the lack of host *Usp47* results in an increased percentage of infiltrating CD8^+^ CTLs in the xenografts, suggesting an enhanced antitumor immune response mediated by CTLs. Conversely, Usp47 deficiency led to a decrease in CD4^+^ Th cells, indicating a potential impairment in the helper T cell-mediated immune response. Further examination of CTL subsets showed increased activated and effector CTLs, as well as exhausted CTLs but a decrease in naïve CTLs in *Usp47*^*−/−*^ mice. These findings suggest that Usp47 may play a critical role in CTL activation. Moreover, our data indicate that deletion of host *Usp47* gene leads to a significant decrease in the proportion of PMN-MDSCs, while the level of M-MDSCs (monocyte MDSCs) increases relatively. PMN-MDSCs primarily contribute to the specific inactivation of antigen-specific T cells by producing high levels of reactive oxygen species (ROS) and directly engaging with T cells through cell-cell contact. On the other hand, M-MDSCs exhibit a different mechanism of immunosuppression. They produce high levels of cytokines such as Tgfβ, Arginase (Arg1), and inducible nitric oxide synthase (iNos), ultimately reducing non-specific T cell responses. Considering the central role of antigen-specific T cell responses in anti-tumor immunity, our finding provides a partial explanation for our understanding of increased activation of tumor infiltrated CTLs in *Usp47*^*−/−*^ mice. Collectively, the altered CTL response within the TME due to Usp47 deficiency could impact antitumor immune responses and contribute to changes in tumor growth dynamics.

## Conclusion

Our study provides valuable insights into the role of USP47 in host anti-tumor immune response. The differential expression patterns of USP47 in various tumors and its association with infiltrating immune cells underscore the significance of its potential as a biomarker for tumor prognosis and as a therapeutic target. Targeting USP47 may offer new avenues for developing novel treatments that modulate the immune response and inhibit tumor growth. Further research is needed to elucidate the underlying mechanisms by which USP47 influences tumor biology and to explore its therapeutic potential in a broader range of cancer types.

### Supplementary Information

Below is the link to the electronic supplementary material.

Supplementary file1 (DOCX 82 KB)

## Data Availability

The data presented in this study are available on request from the corresponding author. The data are not publicly available due to privacy.

## References

[1] Shi J, Liu Y, Xu X, Zhang W, Yu T, Jia J, Liu C (2015) Deubiquitinase USP47/UBP64E regulates beta-catenin ubiquitination and degradation and plays a positive role in wnt signaling. Mol Cell Biol 35:3301–3311. 10.1128/MCB.00373-15 26169834 10.1128/MCB.00373-15 PMC4561727

[2] Yu L, Dong L, Wang Y et al (2019) Reversible regulation of SATB1 ubiquitination by USP47 and SMURF2 mediates colon cancer cell proliferation and tumor progression. Cancer Lett 448:40–51. 10.1016/j.canlet.2019.01.039 30742943 10.1016/j.canlet.2019.01.039

[3] Parsons JL, Dianova II, Khoronenkova SV, Edelmann MJ, Kessler BM, Dianov GL (2011) USP47 is a deubiquitylating enzyme that regulates base excision repair by controlling steady-state levels of DNA polymerase beta. Mol Cell 41:609–615. 10.1016/j.molcel.2011.02.016 21362556 10.1016/j.molcel.2011.02.016

[4] Cho J, Park J, Shin SC, Jang M, Kim JH, Kim EE, Song EJ (2020) USP47 promotes tumorigenesis by negative regulation of p53 through deubiquitinating ribosomal protein S2. Cancers (Basel). 10.3390/cancers12051137 32370049 10.3390/cancers12051137 PMC7281321

[5] Pan B, Yang Y, Li J, Wang Y, Fang C, Yu FX, Xu Y (2020) USP47-mediated deubiquitination and stabilization of YAP contributes to the progression of colorectal cancer. Protein Cell 11:138–143. 10.1007/s13238-019-00674-w 31748975 10.1007/s13238-019-00674-w PMC6954888

[6] Sako-Kubota K, Tanaka N, Nagae S, Meng W, Takeichi M (2014) Minus end-directed motor KIFC3 suppresses E-cadherin degradation by recruiting USP47 to adherens junctions. Mol Biol Cell 25:3851–3860. 10.1091/mbc.E14-07-1245 25253721 10.1091/mbc.E14-07-1245 PMC4244195

[7] Pan K, Fu J, Xu W (2021) Role of ubiquitin-specific peptidase 47 in cancers and other diseases. Front Cell Dev Biol 9:726632. 10.3389/fcell.2021.726632 34604226 10.3389/fcell.2021.726632 PMC8484750

[8] Zhang B, Yin Y, Hu Y, Zhang J, Bian Z, Song M, Hua D, Huang Z (2015) MicroRNA-204-5p inhibits gastric cancer cell proliferation by downregulating USP47 and RAB22A. Med Oncol 32:331. 10.1007/s12032-014-0331-y 25429829 10.1007/s12032-014-0331-y

[9] Lei H, Xu HZ, Shan HZ et al (2021) Targeting USP47 overcomes tyrosine kinase inhibitor resistance and eradicates leukemia stem/progenitor cells in chronic myelogenous leukemia. Nat Commun 12:51. 10.1038/s41467-020-20259-0 33397955 10.1038/s41467-020-20259-0 PMC7782553

[10] Yang SW, Oh KH, Park E et al (2013) USP47 and C terminus of Hsp70-interacting protein (CHIP) antagonistically regulate katanin-p60-mediated axonal growth. J Neurosci 33:12728–12738. 10.1523/JNEUROSCI.0698-13.2013 23904609 10.1523/JNEUROSCI.0698-13.2013 PMC4469866

[11] Tang R, Jin P, Shen C et al (2023) Single-cell RNA sequencing reveals the transcriptomic landscape of kidneys in patients with ischemic acute kidney injury. Chin Med J (Engl) 136:1177–1187. 10.1097/CM9.0000000000002679 37083129 10.1097/CM9.0000000000002679 PMC10278705

[12] Lei H, Yang L, Xu H, Wang Z, Li X, Liu M, Wu Y (2022) Ubiquitin-specific protease 47 regulates intestinal inflammation through deubiquitination of TRAF6 in epithelial cells. Sci China Life Sci 65:1624–1635. 10.1007/s11427-021-2040-8 35235149 10.1007/s11427-021-2040-8

[13] Palazon-Riquelme P, Worboys JD, Green J, Valera A, Martin-Sanchez F, Pellegrini C, Brough D, Lopez-Castejon G (2018) USP7 and USP47 deubiquitinases regulate NLRP3 inflammasome activation. EMBO Rep. 10.15252/embr.201744766 30206189 10.15252/embr.201744766 PMC6172458

[14] Wang A, Huang H, Shi J-H et al (2023) USP47 inhibits m6A-dependent c-Myc translation to maintain regulatory T cell metabolic and functional homeostasis. J Clin Invest. 10.1172/jci169365 37788092 10.1172/jci169365 PMC10688989

[15] Chen HY, Tang RC, Liang JW et al (2023) Deubiquitinase USP47 attenuates virus-induced type I interferon signaling. Int Immunopharmacol 118:110040. 10.1016/j.intimp.2023.110040 37001379 10.1016/j.intimp.2023.110040

[16] De Palma M, Biziato D, Petrova TV (2017) Microenvironmental regulation of tumour angiogenesis. Nat Rev Cancer 17:457–474. 10.1038/nrc.2017.51 28706266 10.1038/nrc.2017.51

[17] Hinshaw DC, Shevde LA (2019) The tumor microenvironment innately modulates cancer progression. Cancer Res 79:4557–4566. 10.1158/0008-5472.CAN-18-3962 31350295 10.1158/0008-5472.CAN-18-3962 PMC6744958

[18] Zhou C, Liu Q, Xiang Y, Gou X, Li W (2022) Role of the tumor immune microenvironment in tumor immunotherapy. Oncol Lett 23:53. 10.3892/ol.2021.13171 34992685 10.3892/ol.2021.13171 PMC8721848

[19] Zhu PF, Wang MX, Chen ZL, Yang L (2021) Targeting the tumor microenvironment: a literature review of the novel anti-tumor mechanism of statins. Front Oncol 11:761107. 10.3389/fonc.2021.761107 34858839 10.3389/fonc.2021.761107 PMC8632059

[20] Li MO, Wolf N, Raulet DH et al (2021) Innate immune cells in the tumor microenvironment. Cancer Cell 39:725–729. 10.1016/j.ccell.2021.05.016 34129817 10.1016/j.ccell.2021.05.016

[21] Gao S, Hsu TW, Li MO (2021) Immunity beyond cancer cells: perspective from tumor tissue. Trends Cancer 7:1010–1019. 10.1016/j.trecan.2021.06.007 34305041 10.1016/j.trecan.2021.06.007 PMC8541902

[22] Anandappa AJ, Wu CJ, Ott PA (2020) Directing traffic: how to effectively drive T cells into tumors. Cancer Discov 10:185–197. 10.1158/2159-8290.CD-19-0790 31974169 10.1158/2159-8290.CD-19-0790 PMC7007384

[23] Vodnala SK, Eil R, Kishton RJ et al (2019) T cell stemness and dysfunction in tumors are triggered by a common mechanism. Science. 10.1126/science.aau0135 30923193 10.1126/science.aau0135 PMC8194369

[24] Zhang L, Romero P (2018) Metabolic control of CD8(+) T cell fate decisions and antitumor immunity. Trends Mol Med 24:30–48. 10.1016/j.molmed.2017.11.005 29246759 10.1016/j.molmed.2017.11.005

[25] Kumar S, Singh SK, Rana B, Rana A (2021) Tumor-infiltrating CD8(+) T cell antitumor efficacy and exhaustion: molecular insights. Drug Discov Today 26:951–967. 10.1016/j.drudis.2021.01.002 33450394 10.1016/j.drudis.2021.01.002 PMC8131230

