# Supplementary material for: USP47 deficiency in mice modulates tumor infiltrating immune cells and enhances antitumor immune responses in prostate cancer
Source: Cancer Immunol Immunother. 2024 Jun 4;73(8):143. doi: 10.1007/s00262-024-03730-5 (PMC11150355; doi:10.1007/s00262-024-03730-5)
Supplement: Supplementary file 1 — Supplementary file1 (DOCX 82 KB) [file 262_2024_3730_MOESM1_ESM.docx]

**Supplemental Materials**

**Fig. S1** Effects of *Usp47* knockout on the apoptosis of CTLs. Early apoptosis: Annexin V^+^/PI^-^, Late apoptosis: Annexin V^+^/PI^+^, necrosis: Annexin V^-^/PI^+^. *p<0.05; **p<0.01.

Fig. S2 Effects of *Usp47* knockout on the proportion of MDSCs.

Table S1. The antibodies were employed in this study.

| Antibodies | Source | Nation | Code number | Application |
| --- | --- | --- | --- | --- |
| anti-CD45-[PerCP-Cyanine5.5,](https://www.thermofisher.cn/antibody/product/CD45-Antibody-clone-30-F11-Monoclonal/45-0451-82) | eBioscience | USA | 45-0451-82 | 0.1ug/10^6^ cells |
| anti-B220-Alexa Flour 405 | Invitrogen | USA | RM2626 | 0.1ug/10^6^ cells |
| anti-CD3-APC | Biolegend | USA | 100235 | 0.1ug/10^6^ cells |
| anti-CD8a-APC-eFluor780 | Invitrogen | USA | 47-0081-82 | 0.1ug/10^6^ cells |
| anti-CD69-eFlour450 | Invitrogen | USA | 48-0691-82 | 0.1ug/10^6^ cells |
| anti-CD44-PE-Cyanine7 | Invitrogen | USA | 25-0441-82 | 0.1ug/10^6^ cells |
| anti-CD62L-APC | Biolegnd | USA | 104411 | 0.1ug/10^6^ cells |
| anti-CTLA4-PE/Cyanine7 | Biolegnd | USA | 106313 | 0.1ug/10^6^ cells |
| anti-PD1-eFluor450 | Invitrogen | USA | 48-9985-82 | 0.1ug/10^6^ cells |
| anti-CD11b-APC | Invitrogen | USA | 17-0112-82 | 0.1ug/10^6^ cells |
| anti-NK1.1-PE | Invitrogen | USA | 25-5941-82 | 0.1ug/10^6^ cells |
| anti-Ly6G/6C (Gr-1)-PE | Invitrogen | USA | 12-5931-81 | 0.1ug/10^6^ cells |
| anti-F4/80-FITC | Invitrogen | USA | 11-4801-85 | 0.1ug/10^6^ cells |
